# Viral-based gene therapy clinical trials for immune deficiencies and blood disorders from 2013 until 2023 - an overview

**DOI:** 10.1016/j.reth.2024.12.007

**Published:** 2024-12-31

**Authors:** Shirin Eshghi, Mahsa Mousakhan Bakhtiari, Maryam Behfar, Elaheh Izadi, Parisa Naji, Leila Jafari, Rashin Mohseni, Zohreh Saltanatpour, Amir Ali Hamidieh

**Affiliations:** a Pediatric Cell and Gene Therapy Research Center, Gene, Cell & Tissue Research Institute, Tehran University of Medical Science, Tehran, Iran; b Stem Cell and Regenerative Medicine Innovation Center, Tehran University of Medical Sciences, Tehran, Iran

**Keywords:** Gene therapy, Viral gene delivery, Viral vectors, Clinical trials, Immune deficiency, Blood disorder, HSCT

## Abstract

Gene therapy (GT) as a groundbreaking approach holds promise for treating many diseases including immune deficiencies and blood disorders. GT can benefit patients suffering from these diseases, especially those without matched donors or who are at risk after hematopoietic stem cell transplantation (HSCT). Due to all the advances in the field of GT, its main challenge is still gene delivery. Generally, gene delivery systems are categorized into two types depending on utilized vectors: non-viral and viral. Viral vectors are commonly used in GT because of their high efficiency compared to non-viral vectors. In this article, all clinical trials on viral-based GT (with the exclusion of CRISPR and CAR-T cell Therapy) in the last decade for immune deficiencies and blood disorders including Severe combined immune deficiency (SCID), Wiskott-Aldrich syndrome (WAS), Chronic granulomatous disease (CGD), Leukocyte adhesion deficiency (LAD), Fanconi anemia (FA), Hemoglobinopathies, and Hemophilia will thoroughly be discussed. Moreover, viral vectors used in these trials including Retroviruses (RVs), Lentiviruses (LVs), and Adeno-Associated Viruses (AAVs) will be reviewed. This review provides a concise overview of traditional treatments for the mentioned disease and precise details of their viral-based GT clinical trial studies in the last decade, then presents the advantages, disadvantages, and potential adverse events of GT. In conclusion, this review presents GT as a hopeful and growing field in healthcare that could offer cures to diseases that were previously thought to be untreatable.

## Abbreviations

SAE: serious adverse event
AE: adverse event
MA: myeloablative
SCID: severe combined immunodeficiency
WAS: wiskott-aldrich syndrome
CGD: chronic granulomatous disease
LAD: leukocyte adhesion deficiency;
Hb: hemoglobin
TDT: transfusion-dependent thalassemia
BM: bone marrow
BU: busulfan
FLU: fludarabine
AAV: adeno-associated virus
LV: lentivirus
RV: retrovirus
HIV: human immunodeficiency virus
ADA: adenosine deaminase
IRT: immunoglobulin replacement therapy
GvHD: graft-versus-host disease
GF: graft failure
HLA: human leukocyte antigen
NK: natural killer
SIN: self-inactivating
TREC: T-cell receptor excision circle
IVIG: intravenous immunoglobulin
Ig: immunoglobulin
ERT: enzyme replacement therapy
FDA: food and drug administration
FDCT: first definitive cellular therapy
ART: artemis-deficient
CMV: cytomegalovirus
PLT: platelet
G-CSF: granulocyte-colony stimulating factor
PB: peripheral blood
UCB: umbilical cord blood
NADPH: nicotinamide adenine dinucleotide phosphate
allo-HSCT: allogenic hematopoietic stem cell transplantation
MAP3K7: mitogen-activated protein kinase kinase kinase 7
ALL: acute lymphoblastic leukemia
AML: acute myeloid leukemia
MDS: myelodysplastic syndrome
eGFP: enhanced green fluorescent protein
ALT: alanine aminotransferase
NAB: neutralizing antibody
CLL: chronic lymphoblastic leukemia
IV: intravenous

## 1 Introduction

Immune deficiencies and blood disorders are groups of inherited diseases that can be life-threatening and fatal [1]. Currently, existing traditional treatments for these disorders are often non-curative and serve merely to mitigate the symptoms. Hematopoietic stem cell transplantation (HSCT) which refers to the transplantation of stem cells from various sources including bone marrow (BM), peripheral blood (PB), and umbilical cord blood (UCB) has long been the only curative approach for the majority of immune deficiencies and blood disorders [[2], [3], [4], [5], [6]]. However, this approach is not completely safe and is associated with complications. One major concern associated with HSCT is finding a competent donor (fully matched). In many cases, lack of a matched donor can amplify the risk of acute or chronic Graft-versus-host disease (GvHD), which is already a risk factor even in those who have undergone HSCT with a fully-matched donor [4,7,8]. Moreover, patients who have undergone HSCT encounter an elevated risk of developing infections, organ toxicity, secondary malignancies, and graft failure (GF). Some people also might not even have a proper donor. As a result, the quest for finding a safer and more effective alternative to previous risky or non-curative treatment approaches is necessary [[2], [3], [4], [5],[8], [9], [10]].

Gene therapy (GT) is a promising medical approach to treat or prevent several incurable diseases such as immune deficiencies and blood disorders that have made great progress in recent years [11]. Nowadays multiple clinical trials are finishing the first phase or entering the second, which are working to demonstrate the safety and efficacy of GT [12]. GT is completely dependent on using a vector as a delivery system to transfer the desired genetic material into the target cells. Due to all the advances in the field of GT, the main challenge in this field is still gene delivery [11]. Typically, gene delivery systems are categorized into two types depending on utilized vectors: non-viral and viral vectors. Non-viral vectors such as polymers, lipids, and inorganic particles are considered to have less cytotoxicity, mutagenesis, and immunogenicity than viral vectors. Nonetheless, these vectors are still under the question of safety, specificity, the duration of gene expression, and gene transfer efficiency [13]. Although viral vectors are associated with risks such as immunogenicity, pathogenicity, and toxicity, they are commonly used in GT because of some superiorities to non-viral vectors such as the higher efficiency and accuracy in gene delivery into target cells, long-term transgene expression, and tissue tropism which leads to optimum efficiency [[14], [15], [16]]. Many types of viruses are being used in GT, e.g., Retroviruses (RVs), Lentiviruses (LVs), Adeno Viruses (Ads), Adeno-Associated Viruses (AAVs), Herpes Simplex Viruses (HSVs), etc. [16].

This review intends to take a brief look at traditional treatments and details of viral-based GT trials for immune deficiencies and blood disorders in the last decade and presents the efficacy, advantages, disadvantages, potential adverse events (AEs), and outcomes of GT. The disorders being reviewed are as follows: Severe combined immune deficiency (SCID); Wiskott-Aldrich syndrome (WAS); Chronic granulomatous disease (CGD); Leukocyte adhesion deficiency (LAD); Sickle cell disease (SCD); β-Thalassemia, Fanconi anemia (FA) and Hemophilia. Moreover, a summary of viral vectors used in these trials including RVs, LVs, and AAVs, is stated. It is to be noted that the concept of CAR-T cell therapy and CRISPR GT is not mentioned in this article. This review triggers the points that can help those concerning the safety, efficacy, and outcome of viral-based GT for immunodeficiencies and blood disorders.

### 1.1 Viral vectors

**Fig. 1:**
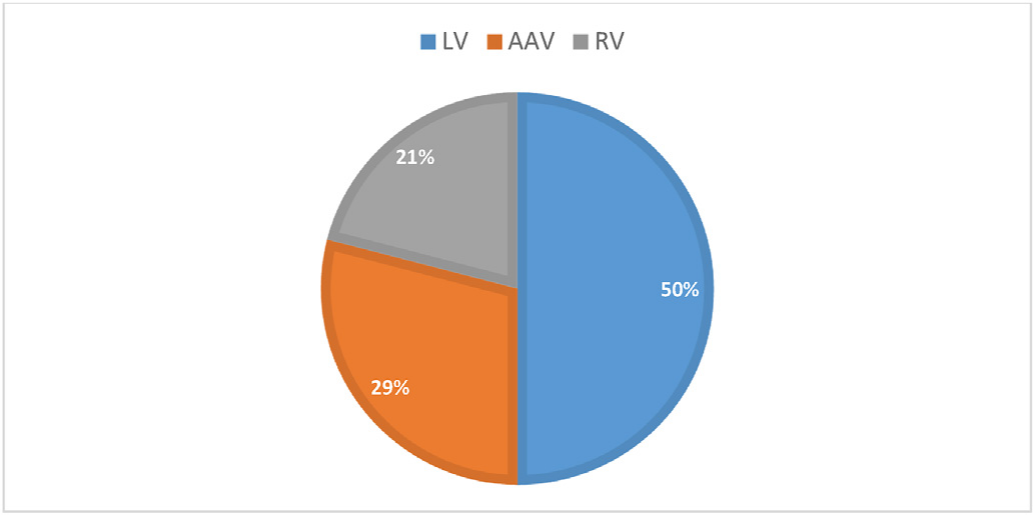
Percentage of the frequency of vectors utilized in the GT trials of immune deficiencies and blood disorders.

Generally, RVs, LVs, AAVs, are the most frequently used viral vectors in GT trials [11]. The five most frequently used viral vectors are going to be further discussed here regarding characteristics and efficacy. Not all these vectors have been utilized in GT trials of immune deficiencies and blood disorders and only LVs, AAVs, and RVs have been used with the utilization percentage illustrated in Fig. 1.

#### 1.1.1 Retrovirus vectors

RVs are a group of single-stranded RNA viruses belonging to the *Retroviridae* family with many features regarding GT. RVs are naturally unique. They can convert their single-stranded RNA into double-stranded DNA with their reverse transcriptase enzyme and integrate into the host genome permanently. The first clinical trial using this virus as a vector was not quite satisfying since, although the overall GT results were favorable, because the vector had an intact long terminal repeat, the patients consequently developed leukemia [17,18]. After that, mediated γ-RVs were introduced, from which most of the viral genome content was deleted to overcome this problem [15]. α-RVs and γ-RVs are two types of RVs that α-RVs have many superiorities to γ-RVs, for instance unlike γ-RVs, α-RVs are capable of infecting both dividing and non-dividing cells. α-RVs have a lower risk of mutagenesis since they can integrate more uniformly within the genome than γ-RVs resulting in lower genotoxicity compared with γ-RV and LV (described further) vectors. However, it seems that the use of α-RVs in trials is limited and the concurrently developed LVs has become mainstream, since no use of this form of RV has been mentioned in the recent decade, instead, many trials have utilized LVs [19]. In terms of genotoxicity, especially in γ-RVs, self-inactivating (SIN) vectors were introduced from which the enhancer elements of the vector are removed [17]. However, in general, γ-RVs have some clinical drawbacks. These vectors can only infect dividing cells, are unable to infect non-dividing cells, and are highly mutagenic. They are capable of integration in the genome randomly and provide long-term expression [20].

#### 1.1.2 Lentivirus vectors

LVs derived from human immunodeficiency virus type 1 (HIV-1) are members of the *Retroviridae* family, *orthoretroviridae* subfamily, and a genus of RVs with single-stranded RNA [15]. LVs have many attractive characteristics, such as long-term expression, low toxicity, and having a wide range of tissue and cells as targets. LVs can also replicate in both dividing and non-dividing cells [17]. All these features make LVs a tempting option for studies requiring long-lasting gene expression. However, LVs can only hold 8 kb of transgene [20]. LVs can also integrate with the host genome which in turn can cause mutagenesis [17]. To improve safety, different generations of LVs have been developed so far, with increasing safety in each generation [21,22]. There are generally four generations of LVs. The first generation of this HIV-derived vector had many HIV genes and the chance of integration into the human genome. To address this risk, a second generation of LVs was introduced which had fewer HIV genes. In the third generation, an interesting feature, SIN was introduced, and the capsid proteins were put into 3 plasmids instead of 2 so that the incidence of re-emerging into wild-type HIV is reduced. The fourth generation was introduced to address homologous recombination, nonetheless, this generation's use is limited [15]. LVs have a broad-spectrum tropism, for instance, the HIV-1 serotype targets CD4-expressing cells primarily, but with the manipulation in this serotype, nowadays it can target human CD34^+^ HSCs. Moreover, pseudotyping with a broad-tropism envelope, such as the vesicular stomatitis virus glycoprotein (VSV-G), has also vastly contributed to the ability of LVs to infect a broad range of cells [23,24]. According to ClinicalTrial.gov, LVs are the most frequently utilized vectors for immune deficiencies and blood disorders.

#### 1.1.3 Adeno-associated virus vectors

AAVs are a family member of Parvovirus belonging to the *Dependovirus* subfamily. AAVs have a single-stranded DNA genome and are named after their members' dependency on other viruses, such as Adenoviruses and Herpes-Simplex viruses for replicating because AAV is a replication-defective virus [15,25]. Nevertheless, in recent clinical research, the so-called helper-free AAVs are being utilized that make AAVs replicate independently [25]. The wild type of AAV (wAAV) exhibits a broad host range, is capable of infecting non-dividing cells, and possesses the characteristic to integrate consistently into a designated location on human chromosome 19 [26]. However, the used recombinant AAV (rAAV) in GT doesn't have the ability to integrate due to the removal of the rep and cap genes from the vector's DNA. Instead, rAAV vectors exist as episomal concatemers in the nucleus of the host cell. In non-dividing cells, these concatemeric forms are stable over the cell's lifespan. Conversely, rAAV DNA is gradually lost during cell division in dividing cells because it does not replicate alongside the cell's DNA. While there is a small chance that rAAV DNA might integrate into the host's genome, such events are extremely rare [27]. AAVs have several attractive characteristics such as low toxicity and pathogenicity. Moreover, AAVs have the unique capability for long-term transgene expression, which is highly advantageous for therapeutic interventions requiring prolonged effects from the transgene. Despite a small packaging capacity (4 kb), AAV's potential to trigger immune responses after repeated administration limits its clinical application [17,20]. Although there are no coding viral sequences in the vector, structural capsid proteins and the transgene nucleic acid sequence and its products can still activate the immune system and cause immunogenicity [14]. Moreover, contrary to the gene transfer mechanism of LVs and RVs that is ex-vivo (harvesting target cells, and transducing them in lab setting) these vectors are capable of in-vivo gene transfer, meaning that they can transfer genes directly in the patients' body [28,29]. Nonetheless, this vector still has less toxicity than other viral vectors. AAV has a broad tissue tropism, in a way that each variant, from 1 to 12, has its specific tropism. For instance, AAV1 is neurotropic and AAV5 can transduce retinal pigmented epithelium and photoreceptors. Almost all AAV variants have muscular tropism, but different variants of AAV can have other tissue tropisms, such as liver, heart, lung, pancreas, etc. [15].

## 2 Immune deficiency disorders

### 2.1 Severe combined immunodeficiency

SCID is a polygenic disease that interferes with the development of natural human T cells. Although this disease is hereditary and antepartum, it can remain asymptomatic at birth [30]. The inheritance is mostly autosomal recessive, except for X-SCID (also designated SCID-X1), which is the most common form of SCID, X-linked, and sporadic. A variety of genes are involved in this disease, such as common gamma chain (γc) of interleukin 2 receptor (IL2RG) in X-SCID, adenosine deaminase (ADA) in adenosine-deaminase-deficient SCIDs (ADA-SCID), and DNA cross-link repair protein 1C (DCLRE1C) in Artemis-deficient SCIDs (ART-SCID) that are the most common types of SCID in GT approaches [2,30,31]. The first appearance of the signs of this disease starts with the early commencement of various infections, growth problems, and malnutrition. The infection commencement of ADA-SCID is earlier than other forms of SCID, but all forms have approximately the same symptoms [2]. In 2021, the birth prevalence of SCID was estimated at 1/100,000 in some parts of the United States and 20/100,000 in the Middle East [32]. This rare disease is important to cure since it can lead to fatal infections and significantly disrupt the lives of affected children and their families [31,32]. Immunoglobulin replacement therapy (IRT), such as intravenous immunoglobulin (IVIG) is prescribed to help patients recover from active infections which is a consequence of their disease [2], and enzyme replacement therapy (ERT) is prescribed for ADA-SCID patients to alleviate their symptoms. However, none of these approaches are curative. For the time being, HSCT is the main promising curative treatment for SCID, but it is associated with risks such as graft-versus-host disease (GvHD) development [8]. There are many clinical trials regarding SCID-GT according to ClinicalTrial.gov but only some of them have published results which are going to be described further. In 2014, Salima Hacein-Bey-Abina et al. reported the results of SIN-RV-GT on 9 SCID-X1 patients. They claimed that the GT had resulted in the resolution of infections, successful engraftment, and immune reconstitution. Moreover, clonal expansions in oncogene sites were less frequent [33] (Table 1, Row No.1). The next year, in 2015, Fabien Touzot et al. published the results of SCID-X1-GT using γ-RV on γc-deficient SCID patients who did not have a matched Human Leukocyte Antigen (HLA) donor. 27 patients were included in this trial and were divided into two groups, one group with 14 patients without a proper donor received γc-mediated hematopoietic stem cell (HSC) without any conditioning regimen (GT group), and a group of 13 patients underwent regular HSCT from a haploidentical donor (HSCT group). The results showed that four patients of the GT group developed leukemia. One patient out of four died because of chemo-resistance development. The number of expired patients was identical in both groups, but the duration of hospitalization due to infection reasons was significantly lower among the GT group. Only one patient among 14 patients in the GT group had GF, while 3 out of 13 patients in the HSCT group had GF. Natural killer (NK) cell counts, although not normal, were higher in the GT group than in the HSCT group. The overall T-cell reconstitution also occurred significantly faster in the GT group in comparison with the HSCT group. With all stated, this team claimed that GT can be a good alternative for HSCT if long-term safety is met [34] (Table 1, Row No.2). Almost a year later, Suk See De Ravin et al. used the SIN-LV vector to treat the same form of SCID (SCID-X1). The ex-vivo SIN-LV-γc mediated CD34^+^ HSCs with a busulfan (BU)-based conditioning regimen were administered to 5 patients aged between 7 and 23 with a history of undergoing haploidentical HSCT. After SIN-LV-based GT the absolute blood count became within normal without the dependency on blood transfusion. Humoral immune response was restored, and the gene-marked immune cells were also increased selectively in all patients. This study proved that the combination of GT and reduced-intensity conditioning (RIC) regimen is safe and helpful for older patients involved in this trial (22 and 23 years old) who have undergone haploidentical HSCT and were further treated with GT [35] (Table 1 Row No.3). After 6 years, Suk See De Ravin et al. updated the results of this trial about those with longer follow-ups. The initial results of the first 8 patients with longer follow-up out of 19 showed no AEs. All 8 patients showed clonal dominance of the high mobility group AT-hook 2 gene (HMGA2), which is the indicator of malignancy risk, in progenitor and myeloid cells. Moreover, the study demonstrated that a cryptic splicing site within the vector increases in CD34+ clones expressing truncated mRNA transcripts. However, it is worth mentioning that the said cryptic splicing can only happen in vectors containing the chicken hypersensitive site 4 (cHS4) insulator, an insulator sequence of the chicken b-like globin gene cluster, in their long terminal repeat (LTR), that can increase the random integration of the vector [[36], [37], [38], [39]] (Table 1, Row No.3). Not long after the first report of this trial, in 2016, the results of another trial held by Maria Pia Cicalese et al. were published. This trial, in which 18 patients with ADA-SCID underwent RV-based GT with a low dose BU conditioning regimen, showed that ex-vivo gene modification of patient-derived HSCs, and then performing an autologous HSCT (HSC-GT), just like former trials, can increase the immune cell counts. T-cells were successfully matured and T-cell receptor excision circle values (TRECs), although below normal, came more than 100 copies/100 ng. Moreover, like the study of Touzot et al., the B-cell count was still low after GT, but a better function was significant in B-cells. 6 patients out of 18 still needed to take IVIG, but 12 patients not only discontinued the use of IVIG but also showed an increase in the serum immunoglobulin (Ig) A and M levels. This study indicated no serious adverse events (SAE) related to GT, although fatty liver and T-cell receptor changes occurred in one patient, and infections in all patients were reported as GT-related AEs [40] (Table 1, Row No.4). In the next year, Kit L. Shaw et al. also stated that γ-RV-based GT for 10 ADA-SCID patients was highly safe in clinical terms. BU-based conditioning regimen was administered prior to γ-RV GT. Except for the oldest, all the included patients were free of ERT, and three of them were also free of IVIG. No serious GT-related AEs and no Leukproliferative disease were evident in patients. Only one patient couldn't regain immune reconstitution and two patients didn't show proper T-cell function, but all patients showed improvement in lymphocyte counts [41] (Table 1, Row No.5). These weren't the only publications related to this trial. Three investigational teams, Aaron R. Cooper et al., Bryanna Reinhardt et al., and Shanna L. White et al. have published the results of this trial during the last decade to indicate the safety of the GT material for SCID patients. In 2017 for example, Aaron R. Cooper et al. also published the results of this clinical trial utilizing either MND-ADA-mediated or GCsapM-ADA-mediated RV for GT. In contrast to the results from Fabien Touzot et al., this study revealed that none of the 15 patients who underwent RV-based GT developed leukemia or dysplasia [42] (Table 1, Row No.5). Shanna L. White et al. focused on two potential long-term complications of this study: the emergence of blood cells with cancerous mutations (Clonal hematopoiesis of indeterminate potential also known as CHIP) and the shortening of telomeres, which are indicators of cell aging. According to this new finding, none of the patients undergone GT showed CHIP mutations and only one patient showed a significant shortening of telomeres in granulocytes. Overall, the results of this trial were mostly favorable [8] (Table 1, Row No.5).

**Table 1:** Viral based-Gene Therapy Clinical Trials for Immune Deficiency from 2013 until 2023, Details.

Row Number	NCT Number	Trial Phase	Vector	Transgene	Indication	Promoter	Included Patients Number	Conditioning Regimen	Vector Dose	Route	Median FU	GT-related SAE	Key Result Summary	Reference Number
1	NCT01410019, NCT01175239, NCT01129544	I/II	SIN-RV	γc	X-SCID	EF1α-F	9	None	(0.25–2.92) Copies/Cell	N/A	29.1	No	Sustained T-cell reconstitution, with six patients demonstrating normal CD3+, CD4+, and CD8+ T-cell levels, IRT was continued, resolved preexisting infections,	[33]
2	N/A	N/A	γ-RV	γc	X-SCID	N/A	27	MAC for the HSCT group and none for the GT group	N/A	N/A	12 yrs for GT group, 6 yrs in HSCT group	T-ALL	Faster T-cell development in the GT group than the HSCT group, higher NK cell counts in the GT group than the HSCT group	[34]
3	NCT01306019	I/II	LV	γc	X-SCID	EF1α-F	5	Non-MAC with BU	N/A	N/A	N/A	No	Selective expansion of gene-marked T, B, and NK cells, humoral immune response restoration, normal absolute blood count. GT with conditioning improves patient outcomes	[35]
							19	6 mg/kg BU					Dominant HMGA2 VIS clones, altering the vector's splice site corrected transduction of mRNA caused by vector's cryptic splice site without losing function.	[36]
4	NCT00598481	II	RV	ADA	ADA-SCID	N/A	18	Low-dose BU	(0.06–2.28) Copies/Cell	N/A	6.9 yrs.	No	Immune reconstitution, less severe infection frequency, improved B-cell function despite low count, IVIG discontinuation, increase in serum IgM and IgA	[40]
5	NCT00794508, NCT00018018	I/II	γ-RV	ADA	ADA-SCID	N/A	10	RIC with BU	(0.001–0.2) Copies/Cell	N/A	N/A	No	Immune reconstitution, ERT discontinuation, IVIG discontinuation, ADA activity normalization	[44]
									(0.1–2.6) Copies/Cell		57 Mos.		ERT discontinuation, IVIG discontinuation, No vector-related complication	[41]
						140 kb ETS2, 285 kb ERG		CRC with BU	N/A		N/A		Correlation of clonal diversity with conditioning regimen intensity	[42]
			LV			N/A		RIC with BU	N/A		44 Mos.		Significant telomere length decreases in one patient	[8]
6	NCT01512888	I/II	LV	γc	X-SCID	N/A	8	Non-MAC with BU	(0.17–1.13) Copies/Cell	N/A	16.4 Mos.	N/A	IgM normalization, Tand B cell reconstitutions, NK cell normalization	[43]
7	NCT01852071, NCT02999984, NCT01380990	I/II	LV	ADA	ADA-SCID	EFS	50	Low-dose BU	(0.25–6.53) Copies/Cell	IV	24 Mos.	Staphylococcus infection	IVIG discontinuation, immune reconstitution, ERT discontinuation	[45]
8	NCT01186913, NCT01346150	N/A	RV and LV	ADA	ADA-SCID	N/A	131	RIC with BU	N/A	N/A	N/A	No	ERT discontinuation, No difference between HSCT and GT in terms of overall survival	[46]
9	NCT03538899	I/II	LV	DCLRE1C	ART-SCID	Endogenous DCLRE1C	10	Low dose	(1.6–3.9) Copies/Cell	N/A	31.2 Mos.	No	T-cell immune reconstitution, immune response normalization	[47]
10	NCT01515462	I/II	SIN-LV	WAS	WAS	1.6 kb reconstituted WAS gene	3	RIC	(1.4–2.8) Copies/Genome		N/A	No	Multi-lineage engraftment lasting up to 30 months in all patients, resolution of eczema and reduced infections, No abnormal cell expansions detected, improved PLT counts, sustained WAS protein expression.	[48]
11	NCT01371981	I/II	γ-RV	WAS	WAS	N/A	10	8 mg/kg BU	(1.7–5.2) Copies/Cell	IV	47.4 Mos.	T-ALL, AML	Sustained engraftment, WAS protein sustained expression, partial or complete improvement of WAS symptoms, including immune deficiencies, autoimmune problems, and bleeding complications	[49]
12	NCT01515462	I/II	LV	WAS	WAS	Endogenous WAS	4	RIC with FLU and BU	(1.4–2.8) Copies/Cell	IV	N/A	N/A	Increased gene marked and normalized B-cell, improvement of Ig levels, autoantibody production, improved eczema	[50]
						1∙6 kb reconstituted WAS	9	RIC with BU FLU	N/A		3.6 yrs.	Gastroenteritis in 2 patients	Ig supplementation discontinuation, immune function development, decrease in severe infection, increase in PLT count, resolved severe bleeding	[52]
							13	Sub-MAC	(0.9–4.3) Copies/Cell		N/A	N/A	Accelerated neutrophil and PLT recovery	[53]
13	NCT01347346, NCT01347242	I/II	LV	WAS	WAS	1.6 kb fragment of the WAS proximal promoter	7	MAC with BU and FLU	(0.6–2.8) Copies/Cell	N/A	27 MO	No	Resolved eczema and infection susceptibility, autoimmunity improvement, discontinuation of thrombopoietic agonists	[51]
14	NCT02234934, NCT01855685	I/II	LV	CYBB∗	X-CGD	CMV	9	MAC with BU	0.7–5.5 Copies/Cell	IV	N/A	Immune reconstitution inflammatory syndrome	Elimination of disease-related infections, discontinuation of CGD-related antibodies	[55]
15	NCT00927134	I/II	γ-RV	CYBB∗	X-CGD	N/A	2	RIC with BU	0.8 Copies/Genome	IV	N/A	MDS	MDS with monosomy 7, resolved invasive *Aspergillus nidulans* in both patients	[57]
16	NCT02757911	I/II	LV	CYBB†	X-CGD	N/A	4	MAC with BU	0.99–1.73 Copies/Cell	IV	2 yrs.	No	Blood cell reconstitution	[58]
17	NCT03812263	I	LV	ITGB2	LAD	N/A	2	MAC with BU	3.8 Copies/Cell	N/A	N/A	No	Rapid neutrophil engraftment, improvements in CD18 expression, resolution of skin lesions	[63]

Mos: months, Yrs.: years, Ig: Immunoglobulin, N/A: not applicable, IV: intravenous, vg: vector genome, kg: kilogram, PLT: platelet SAE: severe adverse event, ERT: enzyme replacement therapy, T-ALL: T-cell acute lymphoblastic leukemia, AML: acute myeloid leukemia, HSCT: hematopoietic stem cell transplantation, RIC: reduced-intensity conditioning, BU: Busulfan, FLU: fludarabine, conditioning, MAC: myeloablative conditioning, FU: follow-up.

∗ CYBB is the gene encoding transmembrane glycoprotein gp91^phox^ on the X chromosome. The mutation in this gene is associated with two-thirds of CGD incidences [41].

In 2019, after two years of silence regarding GT trial results, E. Mamcarz et al. finally published the results of a clinical trial on 8 SCID-X1 pediatric patients without a matched sibling donor. The protocol was approved by the Food and Drug Administration (FDA). This team tested the feasibility of the γc-mediated LV. In this trial's protocol, like the others described former, a low-dose BU was administered to the patients as a conditioning regimen. The results indicated that γc-mediated LV-GT can bring NK cells within normal range unlike other trials and bring immune reconstitution of T and B cells. All patients except one, were free of IVIG injection. Only one SAE related to BU toxicity was observed and no GT-related AE was evident [43] (Table 1, Row No.6). ADA-SCID GT in 2021 didn't come with a significant change either. In 2021, the results of two other trials held by Bryanna Reinhardt's [44] and Donald B. Kohn's teams [45] were published regarding ADA-SCID GT using different vectors, both of which showed a feasible effective response, although the number of patients in each trial differed. Kohn's trial utilized LVs, while Reinhardt's team (which updated the trial initially held by Kit L. Shaw et al.) opted to utilize RV for their approach, nonetheless, both trials had BU-based conditioning regimens. None of these two trials reported evidence of leukoproliferative disease. ERT for most patients in both trials was discontinued and IRT was discontinued for almost all patients [44,45] (Table 1, Row No.5,7). 2022 was a year full of GT trials on SCID patients with four published papers of demonstration, most of which were performed on ADA-SCID [8,36,46,47]. In one of the trials conducted this year, Geoffrey D. E. Cuvelier et al. included 131 ADA-SCID patients and divided them into four groups. Those who only received hematopoietic cell transplant (First group), those who received ERT prior to HSCT (Second group), those who received ERT then underwent GT (Third group), and those who only received ERT with no succeeding operation (Fourth group). In this trial, both LV and RV were considered to treat the third group. Notably, the frequency of autoimmunity didn't differ among first, second, and third (GT) groups during the first definitive cellular therapy (FDCT). Event-free survival of the group undergoing GT (third group) was better than the first group, but the results didn't differ between the GT and second groups. No difference in infection recovery was observed between first and GT groups and the overall survival of both first and GT groups was equal (100 percent). Immune reconstitution improvement was similar in LV-based GT and second group, and the RV-based GT showed less T-cell count. Moreover, 4 patients out of 12 who had RV-based GT were back on ERT again after GT, and none of 21 LV-based GT had this matter. As a conclusion, the authors stated that if a patient doesn't have a matched related donor or the GT is not an option for him, alternative donor hematopoietic cell transplant might be viable to pursue [46] (Table 1, Row No.8). In the last trial published in 2022, Morton J. Cowan et al., in a departure from other trials of this decade, initiated GT on ART-SCID patients. They arranged an ex-vivo DCLRE1C-mediated LV-based GT on 10 pediatric patients who did not have a matched sibling donor. BU was administered for this cohort as a conditioning regimen. As a result of LV-based GT, all patients had immune reconstitution. B-cell count was also normalized in four patients of those followed for 24 months, and three of them had normalized immune responses. A patient infected with cytomegalovirus (CMV) required an additional dose of DCLRE1C-mediated LV HSCs to enable clearance of the infection within the body. Overall, the results of this trial proved that LV-based GT on ART-SCID pediatric patients can provide functional T and B-cells, although there were GT-related AEs [47] (Table 1, Row No.9). In summary, based on the extensive data from these trials, it can be concluded that GT with viral vectors has generally been promising. If SAEs become less common, this approach could potentially become the best approach for treating SCID patients.

### 2.2 Wiskott - Aldrich syndrome

WAS is one of the X-linked primary immune deficiencies with a mutation in the WAS protein (WASP) gene, which can cause eczema, thrombocytopenia, autoimmune problems, recurrent infections, increased risk of lymphoma, and low platelet (PLT) count. The prevalence of this disease is roughly between 1 in 50,000 and 1 in 250,000 live births. A moderate form of WAS is X-linked thrombocytopenia. The current treatment for WAS is HSCT, but GT is making its way as an effective treatment for WAS [3]. The first WAS-GT in the current decade was performed by Aiuti et, al. in Italy. This group utilized ex-vivo GT using LV vectors on three patients. The results of this trial were favorable, with no integration of LVs near oncogenes in the genome, improved PLT counts and immune response. Moreover, all patients experienced engraftment. Nonetheless, two patients showed SAEs that claimed not to me relevant to the GT procedure [48] (Table 1, Row No.10). Christian Jörg Braun et al. also reported the results of their trial on WAS a year later, in 2014. This clinical trial was performed on 10 patients with severe WAS, undergoing ex-vivo GT using RV (CMMP-WASP) with a BU-based conditioning regimen. After GT, only one patient had GF. Almost all patients showed corrected PLT, lymphoid, and myeloid cell counts. This trial also proved that GT could treat characteristics associated with WAS immune deficiency, autoimmune conditions, and blood coagulation disorders. Unfortunately, 7 patients developed different kinds of leukemia, thus in general, although GT was a proven feasible and constructive approach for WAS treatment, it can accelerate the risk of leukemia morbidity as a result of using RV [49] (Table 1, Row No.11). In 2015 two ex-vivo LV-based GT trials were held with low-dose BU and Fludarabine (FLU) conditioning regimen in two different countries, by Maria Carmina Castiello et al. in Italy [50], and Salima Hacein-Bey Abina et al. in France [51]. The immune reconstitution in the trial held in Italy was more favorable with an improved function in T, B, and NK cells, as well followed by PLT recovery. Moreover, it is evident that the trial held in Italy demonstrated better gene transfer efficiency in comparison with the French trial. Nonetheless, both cohorts showed a good response to GT with no evidence of leukemia. All patients of both cohorts were alive except one in France which expired due to a previous Herpes-Simplex Virus infection the patient had before LV-GT. All surviving patients in both cohorts were eczema-free and experienced increased Ig levels, yet not all could achieve the normal level. Finally, both trial teams stated that LV-based GT benefits WAS patients [50,51] (Table 1, Row No.12,13). In 2019 Francesca Ferrua et al. updated the results of the trial held in Italy which indicated that overall survival was 100 %, and 7 out of 8 patients were IRT-independent. The frequency of disease-related severe infections also decreased in the patients. There was a significant increase in PLT count which made patients PLT transfusion-free. They also experienced less severe bleeding after GT. In conclusion, this study could illustrate the benefit of GT for WAS and could prove that it can be a good alternative for WAS patients who don't have a full-matched donor [52] (Table 1, Row No.12). For three years no trial outlined the results of GT on WAS, but finally in 2022, Serena Scala et al. came up with a new finding of LV-based GT on WAS that was a continuation of Maria Carmina Castiello et al.’s and Francesca Ferrua et al.’s work. They evaluated the capability of ex-vivo LV-based GT using autologous stem and progenitor cells from two different sources, BM and PB mobilized by Granulocyte-Colony Stimulating Factor (G-CSF). The results proved that neutrophil and PLT engraftments were faster in patients with PB stem cells. The transduction percentage was quite similar in both sources though. Thus, they concluded that the difference between the results of LV-based GT using two stem cell sources is irrelevant to the functional characteristics of the cell source and is more a result of the difference in cell composition [53] (Table 1, Row No.12). Overall, LVs and RVs are the vectors utilized in WAS GT. LV-based GT was successful in all the trials and viral-based GT for WAS is considered feasible and safe.

### 2.3 Chronic granulomatous disease

CGD is a life-threatening member of primary immune deficiencies with a deficiency in nicotinamide adenine dinucleotide phosphate (NADPH) oxidase enzyme. Due to the impaired immune system, patients with CGD are at an increased risk of various infections [4]. CGD can also cause inflammatory diseases, such as granuloma formation [54]. HSCT is a promising curative treatment for CGD, akin to its role in successfully treating two other immune-deficiency conditions, SCID and WAS [4]. The initial RV-based GT trials conducted on CGD patients were carried out without any conditioning regimen, but unfortunately, they did not lead to long-term neutrophil and PLT engraftments, thereby failing to provide tangible benefits to the patients. Additionally, some patients developed leukemia after GT utilization [4,17,55,56]. Nonetheless, in the current decade, three trials that were performed on CGD patients using LVs instead of RVs provided favorable results. In 2020, Donald B. Kohn and his team, which proved the safety of SCID-GT, performed another ex-vivo LV-based GT on CGD and proved that using this approach can safely eliminate the risk of infections related to CGD. Kohn's group offered strong evidence supporting CGD-GT, countering historical viral vector-based GT failures [55] (Table 1, Row No.14). In 2015, Ulrich Siler and his colleagues conducted another GT trial using γ-RV on 2 young males with X-linked CGD. The procedure of this trial is unique since an allogenic HSCT (allo-HSCT) was performed on patients after GT. In the published results of this trial, it is stated that after γ-RV-based GT, patient 1 received allo-HSCT from his HLA-identical sibling, and patient 2 underwent HSCT from an 11/12 HLA-mismatch donor for his first allo-HSCT. After GT, both patients completely recovered from invasive spinal *Aspergillus nidulans* infections that they had prior to GT. Moreover, patient 2 developed myelodysplastic syndrome (MDS), but after the second allo-HSCT from a 9/12 mismatch donor, he fully recovered. The authors stated that allo-HSCT was essential for patients to survive after γ-RV-based GT and SIN-LVs might be a safer way to be utilized in GT for this disease [57] (Table 1, Row No.15). At the time of writing this review, another ex-vivo LV-based GT trial held by Steicy Sobrino et al. showed no proof of any AEs, except one related to a myeloablative (MA) conditioning regimen. 5 patients enrolled in this trial with X-CGD, four of whom received autologous HSC-LV-based GT. After the procedure, all patients experienced blood cell reconstitution, two of 4 patients had engraftment while two others experienced GF. Noteworthy, those who experienced GF had significantly fewer HSCs [58] (Table 1, Row No.16). The results of these three trials altogether proved that LV-based GT with the cooperation of HSC protection treatments can alleviate the symptoms of CGD [[54], [55], [56]]. To sum up, the LV-based GT for CGD patients is safe and favorable providing blood cell reconstitution and infection resolution. The trials didn't have SAE related to GT, except immune reconstitution inflammatory syndrome and MDS which was successfully cured. It is also worth mentioning that in two trials, the use of a myeloid-specific promoter seems to play a more pivotal role than the use of LV as a vector.

### 2.4 Leukocyte adhesion deficiency

LAD is a rare autosomal recessive immune deficiency in bovine, canis, and humans that can cause a delay in umbilical cord separation, recurrent infections, and delayed wound healing [[59], [60], [61]]. There are three different subtypes of LAD; LAD-I, LAD-II, and LAD-III among which LAD-I is the most common subtype [62]. LAD-I is characterized by the absence of the β2-integrin (ITGB2), also called CD18, on leukocytes [10,63]. The prevalence of this disease is 1 in a million [64]. LAD-I patients often experience a high frequency of skin and mucosal infections, inflammation, and intense gingivitis. However, these conditions have demonstrated improvement when treated with Ustekinumab, a monoclonal antibody that inhibits the action of IL-12 and IL-23. HSCT has proven to be an effective treatment for patients with LAD including LAD-I [8,10,36]. Back in 1992, a GT trial utilizing γ-RV was performed on LAD-I patients without any conditioning regimen. This trial didn't benefit the patients clinically and the engraftment didn't occur. Later experiments in a Canine model with LAD-I found that a healthy BM from a sibling, or autologous HSC-GT, can stop infections if given after a non-MA conditioning regimen [10]. In 2020, an abstract detailing a phase I LV-based GT for LAD-I patients was published. This trial indicated that LV-based GT with a MA conditioning regimen has no severe AEs related to the GT and can increase the neutrophil count in both patients involved in the trial. The key benefit of this trial was that the skin lesions were healed and an increased expression of CD18 was observed. To wrap it up, LV-based GT along with a non-MA conditioning regimen might be safe and work for treating serious LAD-I. Donald B Kohn and his colleagues, who started this trial, are now starting to sign up patients for the Phase II trial [63] (Table 1, Row No.17).

## 3 Blood disorders

### 3.1 Hemoglobinopathies

Hemoglobinopathies are a group of common inherited disorders with mostly autosomal recessive inheritance. The prevalence of this group of diseases is estimated to be 7 % of the population around the world. SCD and β-Thalassemia are the most common forms of hemoglobinopathies. HSCT was the only curative approach for both SCD and *β-*Thalassemia during the last few decades, but with the progression of GT, the hopes to find a less aggressive treatment for hemoglobinopathies started to come along [5,6,9]. In the last decade, many trials were initiated regarding viral-based GT of SCD and *β-*Thalassemia, all of which utilized LVs. The brief details of each trial are illustrated in Table 2. In 2015, one GT published by Marina Cavazzana et al. was performed on both SCD and Thalassemia patients utilizing the same GT product. In this trial, 1 patient with SCD and 3 patients with β0/βE-Thalassemia received LV vector encoding βA-T87Q-globin. The results of this trial indicated that there were no AEs related to the GT product. However, the SCD patient produced 51.5 % anti-sickling hemoglobin (HbS) with no complications related to SCD. Moreover, all patients discontinued chronic blood transfusions in different periods after GT [65] (Table 2, Row No.2).

**Table 2:** Viral based-Gene Therapy Clinical Trials for blood disorders from 2013 until 2023, Details.

Row Number	NCT Number	Trial Phase	Vector	Transgene	Indication	Promoter	Included Patients Number	Conditioning Regimen	Vector Dose	Route	Median FU	GT-related SAE	Key Result Summary	Reference Number
1	NCT00430118, NCT01117441	III	AAV	MAP3K7	N/A	327	1 × 10∗4–5 × 10∗5 vg/kg	N/A	N/A	N/A	N/A	N/A	MAP3K7 deletion in about 10 % and point-mutated in about 1 % of children,	[99]
													MAP3K7 deletions are linked to mature immunophenotype, but not treatment response and outcome	
													Depletion of MAP3K7 expression in T-ALL cell lines slowed proliferation and induced apoptosis without affecting NF-κB signaling or target gene expression after TNF-α stimulation.	
2	N/A	N/A	LV	BB305∗	SCD, β-thalassemia	N/A	1 SCD 3 β-thalassemia		0.8–2.1 copies/?	N/A	N/A	No	Production of anti-sickling hemoglobin, chronic transfusion discontinuation in both SCD and β-Thalassemia patients	[65]
3	N/A	I/II	LV	BB305∗	SCD	N/A	7	MAC with BU	0.3–1.3 Copies/Diploid genome	N/A	7.1 Mos.	No	Sustained HbA^T87Q expression^, a decrease in VCN led to a decrease in hb expression	[69]
4	NCT03282656	I	LV	BCH-BB694∗∗∗∗	SCD	N/A	6		1.8–6.2 Copies/Cell	N/A	18 Mos.	No	HbF normalization and proper distribution, RBC transfusion discontinuation	[67]
5	NCT02140554	I/II	LV	BB305∗	SCD	N/A	35	MAC with BU	2.3–5.7 Copies/Cell	N/A	17.3 Mos.	No	Hb level improvement, hemolysis marker reduction	[68]
6	NCT01745120 NCT02151526	I/II	LV	β-globin	β-Thalassemia	CMV	22	MAC with BU	∼1–2 Copies/Cell	IV	26 Mos.	No	HbA^T87Q^ level progression, RBC transfusion discontinuation in non–β0/β0 patients, transfusion frequency reduction in other patients, correction of dyserythropoiesis markers in patients with near-normal hb levels	[70]
							1				3.5 yrs.	N/A	RBC transfusion discontinuation, HIV infection	[71]
7	NCT02453477	I/II	LV	β-globin	β-Thalassemia	CMV	9	MAC with treosulfan–thiotepa	0.10–1.97 Copies/Cell	IB	18 Mos.	N/A	Red-cell transfusion discontinuation or reduction, successful engraftment, rapid recovery of hematopoiesis across multiple lineages	[72]
8	NCT00336362 NCT01206075	N/A	LV	β-globin	β-Thalassemia	N/A	46	N/A	N/A	N/A	N/A	N/A	No difference in engraftment potential between fresh and up to two times freeze/thawed CD34 cells, No transduction rate difference, reduced clonogenic potential in cryopreserved cells with multiple freeze/thaw cycles in cultured but not unmanipulated cells	[73]
9	NCT01639690	I	LV	β-globin	β-Thalassemia	Human β-globin	4		0.01–0.11 Copies/Cell	N/A	90 Mos	No	RBC transfusion reduction in two patients	[74]
10	NCT02906202	III	LV (betibeglogene autotemcel)	β-globin	β-Thalassemia	N/A	23	MAC with BU	N/A	IV	29.5 Mos.	Thrombocytopenia in one patient	Transfusion discontinuation	[75]
11	N/A	I/II	LV	FANCA	FA	N/A	22	Mild	N/A	N/A	N/A	N/A	Anticipated to be a novel treatment for individuals without a compatible donor for HSCT.	[78]
12	NCT01331018	I	LV	FANCA	FA	PGK	3	N/A	0.33–1.83 Copies/Cell	N/A	N/A	No	Gene transfer efficiency and cell viability preservation, >90 % of lineage + cells depletion, CD34^+^ cells (≥60 %) preservation	[79]
13	NCT03157804	I/II	LV	FANCA	FA	N/A	4	N/A	0.17–0.53 Copies/Cell	N/A	N/A	No	Genotoxic integrations and acquired resistance to DNA cross-linking agents., LV-based GT in FANCA patients halted BM failure progression without a prior conditioning regimen	[80]
14	NCT00979238	I	AAV8	Human FIX	Hemophilia B	N/A	10	N/A	Low dose (2 × 10^11^ vg/kg)/Intermediate (6 × 10^11^ vg/kg)/High dose (2 × 10^12^ vg/kg)	IV	N/A	N/A	Circulating FIX level increase, bleeding rate, and factor administration reduction	[82]
15	NCT02576795, NCT03370913	I/II	AAV5	Human FVIII	Hemophilia A	Liver-specific	9	N/A	Low dose (6 × 10^12^)/Intermediate dose (2 × 10^13^)/High dose (6 × 10^13^) vg/kg	IV	52 weeks	Chronic arthropathy	Normalized FVIII activity in high-dose cohort, bleeding rate decrease, FVIII activity in low-dose cohort remained less than 1 IU/dL, low but stable FVIII activity level in intermediate-dose cohort	[83]
							45	N/A	6e13 or 4e13 vg/kg		2 or 3 yrs.	N/A	Transgene-produced FVIII-SQ shows higher activity in OS assays than in CS assays,	[84]
													Transgene-produced FVIII-SQ accelerates early factor Xa and thrombin formation, potentially explaining the higher OS activity	
							15	N/A	Low dose (6 × 10^12^)/Intermediate (2 × 10^13^)/High dose (6 × 10^13^) vg/kg		3 yrs.	ALT elevation, pyrexia with myalgia	Resolved bleeding, F administration reduction	[100]
16	NCT02484092	I/IIa	SPK-9001∗∗ (AAV-Spark100)	Padua FIX	Hemophilia B	Liver-specific	10	N/A	5 × 10^11^ vg/kg	IV	52 weeks	No	Bleeding rate decrease, FIX administration discontinuation, sustained FIX with a mean steady-state activity of 33.7 ± 18.5 %.	[85]
17	NCT02396342	I/II	AAV5	Human FIX	Hemophilia B	Liver-specific	10	N/A	Low dose (5 × 3 10^12^), high dose (2 × 3 10^13^) vg/kg	IV	N/A	ALT elevation, fever	Endogenous FIX activity, FIX administration reduction, bleeding rate decrease	[86]
18	NCT00515710	N/A	AAV2	Human FIX	Hemophilia B	Tissue-specific	7	N/A	8 × 10^10^–2 × 10^12^ vg/kg	IVUS	N/A	No	Persistent increased AAV-neutralizing antibodies to AAV2, AAV5, and AAV8 were observed for up to 15 years post-vector administration, No sustained hepatic toxicity or hepatocellular carcinoma was found during the FU time	[87]
19	NCT01687608	I/II	AAV8	Padua FIX	Hemophilia B	Liver-specific transthyretin	7	N/A	Low dose (2.0 × 10^11^)/Intermediate (1.0 × 10^12^)/High dose (3.0 × 10^12^) vg/kg	N/A	4 yrs.	No	Measurable FIX expression in 7 of 8 participants; peak activity ranged from 32.0 % to 58.5 %, sustained ∼20 % FIX activity for 4 years in one patient; others did not sustain beyond 5–11 weeks	[89]
20	NCT03003533 NCT03432520	I/II	AAVLK03 (SPK-8011)	Human FVIII	Hemophilia A	Liver-specific, truncated transthyretin	18	N/A	Low dose (5 × 10^11^)/High dose (2 × 10^12^) vg/kg	N/A	36.6 Mos.	ALT elevation	Maintained FVIII expression, the bleeding rate decreased	[90]
21	NCT03641703	I/II	AAVS3∗∗∗	Padua FIX	Hemophilia B	FRE1	10	N/A	Low dose 3.84 × 10^11^/Intermediate dose (6.40 × 10^11^)/High dose (8.32 × 10^11^)/Maximum dose (1.28 × 10^12^) vg/kg	IV	27.2 Mos.	ALT elevation/arteriovenous fistula	Dose-dependent increases in FIX levels post-infusion,	[91]
													Sustained activity in 9/10 patients; one resumed prophylaxis,	
													Five patients achieved normal FIX levels, three had subnormal levels	
22	NCT04135300	I	AAV843	Padua FIX	Hemophilia B	Liver-specific	22	N/A	5 × 10^12^ vg/kg	IV	58 weeks	No	Mean FIX coagulation activity (FIX:C) Increased to 36.9 IU/dL, antibodies against vector capsid development, maintained normal liver enzyme levels (ALT and AST) in 80 % of patients, concurrent decrease in FIX coagulation activity, but the level remained above 7 IU/dL	[92]
23	NCT03370913	III	AAV5	Human FVIII	Hemophilia A	Liver-specific	134	N/A	6 × 10^13^ vg/kg	IV	60.2 Mos.	ALT elevation	Mean FVIII activity increased by 41.9 IU/dL at 1 year,	[93]
													FVIII concentrate use and bleeding decreased by 98.6 % & 83.8 %.	
								N/A			5 yrs.	No	FVIII activity maintained for 2 years post-infusion,	[94]
													84.5 % reduction in mean annualized bleeding rate,	
													1.0 expected joint bleeds per year at 5 IU/dL FVIII level	
24	NCT03569891	III	AAV5	Padua FIX	Hemophilia B	N/A	54	N/A	2 × 10^13^ vg/kg	N/A	5 yrs.	No	Bleeding rate reduced by ∼64 %,	[95]
													FIX activity increased by ∼36 % at 6 and 18 months post-treatment,	
													FIX concentrate use decreased by ∼248,825 IU/year/participant	

Mos: months, Yrs.: years, N/A: not applicable, IV: intravenous, IC: intracerebral, IB: intra bone, IVUS: intravascular, PGK: phosphoglycerate kinase, vg: vector genome, kg: kilogram, EF1α-F: a fragment of EF1α, SAE: severe adverse event, HbF: hemoglobin F, FIX: factor IX, FVIII: factor VIII, VCN: vector copy number, HSCT: hematopoietic stem cell transplantation, RIC: reduced-intensity conditioning, BU: busulfan, FU: follow-up, CRC: cytoreductive conditioning, MAC: myeloablative conditioning, HbA^T87Q^: Therapeutic HbA, OS: the one-stage clot, CS: chromogenic-substrate.

Note: Regarding hemophilia GT, some trials have stated that liver enzyme elevations were considered SAE, while some others stated that although liver enzymes elevated, the study did not have SAE. The reason might be the scale of elevation or the baseline of the patient.

∗ BB305 contains a modified version of the human β -globin gene, which has a precise alteration (AT87Q). This change aims to replicate the protective effects against sickling that are found in the Hb of fetuses, known as γ-globin, and can have rapid clinical improvement and provide near-normal total Hb [68,70].

∗∗ SPK-9001 is a recombinant AAV vector comprised of a genetically modified capsid (AAV-Spark100) and an expression cassette that includes the hepatic-control region of the apolipoprotein E gene (APOE) [71].

∗∗∗ AAVS3 is an AAV with a synthetic capsid and can transduce more liver cells [78].

∗∗∗ ^∗^BCH-BB694 is a third-generation self-inactivating lentiviral vector used for mediating erythroid-specific knockdown of BCL11A through RNA interference. It was optimized for therapeutic use, and in preclinical studies, it demonstrated potent induction of HbF in CD34+ cells derived from donors with sickle cell disease [67,101].

The trials for thalassemia and sickle cell disease specifically are showcased in subsequent sections.

#### 3.1.1 Sickle cell disease

*SCD* is one of the most common severe monogenic disorders in the world. This disease is associated with recurrent acute illnesses, hemolytic anemia, progressive organ damage, and detecting abnormal HbS on electrophoresis [66]. A significant amount of fetal Hb (HbF) consisting of α-globins and γ-globins might improve disease symptoms by reducing the formation of HbS polymers and the sickling of red blood cells (RBC) but these approaches are not curative and the only option for SCD patients had been HSCT before the introduction of GT [5,67]. Recent studies indicate that GT is a good alternative for HSCT in SCD patients and a lot of trials were held to justify the applicability and efficacy of SCD-GT [[67], [68], [69]]. In a GT trial held by Julie Kanter et al., in 2016, 7 patients with SCD were treated with autologous HSC-GT with LV encoding BB305. BB305 contains a modified version of the human β-globin gene, which has a precise alteration (A^T87Q^). In this trial, all patients underwent BM harvest for autologous HSC-GT and received a BU-based conditioning regimen. The results of this study indicated that after LV-based GT, the modest expression of HbA^T87Q^ was observable. However, this expression dropped after the decrease in the vector copy number (VCN) in PB [65,69] (Table 2, Row No.3). In the trial published in 2021 by Erica B. Esrick et al., the MA conditioning regimen along with autologous HSC-GT with a LV vector encoding shRNA targeting BCL11A (Inhibitor of γ-globin expression and HbF production in human erythrocytes) was administered to 6 patients. All patients had engraftment and no SAEs associated with GT were reported. There was no evidence of a vaso-occlusive crisis, acute chest syndrome, or stroke after GT and 2 patients were free of the RBC transfusion after engraftment [67] (Table 2, Row No.4). With a one-year interval, in 2022, J. Kanter et al. evaluated LV-based GT encoding β-globin (LentiGlobin) on SCD patients. The results of this trial indicated that by using an MA conditioning regimen along with GT, SCD patients can experience reduced hemolysis markers and increasing HbA^T87Q^ levels. No SAE was associated with GT products. Moreover, no signs of hematologic cancers were observed after three-years follow-up. Although, two cases were diagnosed with AML, since there were no signs of insertional oncogenesis in either case, the AML is assumed not to be relevant to the LentiGlobin administration [68] (Table 2, Row No.5).

#### 3.1.2 β-Thalassemia

More than 200 mutations in the β-globin gene (HBB) are associated with β-Thalassemia, which is a disease caused by abnormal synthesis of β-globin. This form of hemoglobinopathy has been one of the most interesting forms for GT trial initiation [5,6]. In the latest published results of a GT clinical trial for transfusion-dependent β-Thalassemia (TDT) patients which was reported in 2018, A.A. Thompson et al. reported that the utilization of LentiGlobin yielded favorable outcomes. These include the discontinuation of RBC transfusion in non–β0/β0 (homozygous for β-globin gene) patients, a reduction in transfusion frequency in other patients, and correction of dyserythropoiesis biological markers in patients with near-normal Hb levels. No SAE related to GT was noted in this report [70] (Table 2, Row No.6). Once more in 2021, the reliability of Thompson's trial team was reaffirmed through the release of the latest findings of this trial by Suradej Hongeng. As with the previous report of this trial, it was demonstrated that betibeglogene autotemcel, which is an approved LV-based GT product for TDT patients could provide independence to RBC transfusions. However, the focus of this report is that after LV-based GT, one patient was diagnosed with wild-type HIV. This was the first time a wild-type HIV infection was observed in the history of LV vectors, although the authors didn't briefly state that this incidence was because of the GT products [71] (Table 2, Row No.6). In 2018, another trial published by Sarah Marktel et al., utilizing LV-based GT on 9 patients with TDT by a MA conditioning regimen also reported the same findings in adults and children [72] (Table 2, Row No.7). Garyfalia Karponi et al. revolutionized the investigation into potential disparities between the outcomes of LV-based autologous HSC-GT by the fresh CD34^+^ cells and cryopreserved CD34^+^ cells. The results of this intriguing trial, which was published in 2018, were indicative of the comparable efficacy of transduction between fresh cells and cryopreserved cells with one or two freeze/thaw cycles. However, it was noted that cryopreserved cells with multiple freeze/thaw cycles exhibited reduced clonogenic potential [73] (Table 2, Row No.8). Up until 2022, every published trial used a MA conditioning regimen, but in 2022, Farid Boulad et al., modified the conditioning regimen into a RIC regimen. Although the conditioning regimen changed, the results were still favorable in that the RBC dependency was reduced without any SAE associated with the conditioning regimen [74] (Table 2, Row No.9). In the same year, a trial using betibeglogene autotemcel (beti-cel) along with a MA conditioning regimen on 23 TDT patients, led to achieving independence from RBC transfusions in 91 % of the patients. However, 2 patients experienced thrombocytopenia which is possibly related to beti-cel [75] (Table 2, Row No.10). With all the data collected during these trials, LV-based GT for TDT patients is feasible and progressive. However, because of the transformation of the rLV to wild-type HIV during the last decade, further investigation might be needed to give a brief answer about LV-based GT's safety.

### 3.2 Fanconi anemia

FA is a monogenic inherited disorder mostly associated with BM failure that can elevate the risk of cancers, Acute myeloid leukemia (AML), Myelodysplastic syndrome (MDS), and Solid tumors, specifically squamous cell carcinomas in the affected patients. This disease is characterized by chromosomal instability and inter-strand crosslinks repair deficiency. The prevalence of the disease is 1–5 cases per million. Birth defects, café au lait (skin hyperpigmentation) spots, renal complications, and microphthalmia are the common complications associated with FA. The mutation in any of the 23 FANC genes can cause FA. The inheritance is mostly autosomal recessive with the exclusion of FANCB and RAD51/FANCR mutations which are X-linked and dominant negative respectively. HSCT has long been the most promising approach regarding treating BM failure in FA patients, however, with the emergence of GT, it has started to be seen as a viable alternative and is now being utilized in recent clinical trials [76,77]. In a recent LV-based GT trial summary, which was published in 2015, Bent Jakobsen et al. claimed that autologous FANCA-mediated CD34^+^ infusion is expected to provide a new therapeutic approach for FA patients who might not have a properly matched sibling donor [78] (Table 2, Row No.11). A 2018 trial by Jennifer E. Adair et al. found that the main obstacle to FANCA deficiency GT is the low number of CD34^+^ cells and the varied ratios of CD34^+^ expression in FA patients. Only cells with high expression of CD34^+^ are capable of regeneration in a lab setting. Therefore, Adair and her colleagues set up a protocol to deplete lineage^+^ (CD3^+^, CD14^+^, CD16^+^, and CD19^+^) cells from blood and BM sources to purify CD34^+^ cells for repopulation. In this trial, LV vectors were utilized to introduce genetic material into healthy donor cells, carrying either GFP as a control or the complete FANCA cDNA. The outcome of the trial demonstrates that the developed process successfully reduces unwanted (lineage^+^) cells while preserving the majority of the CD34^+^ cells, maintaining the efficiency of gene transfer and cell viability. Moreover, in this study, BM products from healthy donors were separated into two aliquots: one was lineage-depleted, while the other was CD34-enriched. Both resulting cell populations were then exposed to the same LV vector under the same conditions and transferred into immunodeficient mice at equivalent CD34^+^ cell levels. The result of this attempt showed that the transduced lineage^–^ cell products had similar engraftment potential to purified CD34^+^ cells when xenotransplanted at matched CD34^+^ cell doses [79] (Table 2, Row No.12). In another trial published by Paula Río et al., in 2019, it is demonstrated that LV-based GT in patients with a mutation in FANCA without conditioning regimen stemmed the BM failure progression. Moreover, its safety was proven because none of the involved patients developed any GT-related SAE [80] (Table 2, Row No.13).

### 3.3 Hemophilia

Hemophilia is one of the inherited disorders that can interfere with the proper clotting of the human blood. Hemophilia A and B are two forms of this disease with the defective coagulation factors VIII and IX (FVIII and FIX) and its incidence is approximately 1 in 5000 and 1 in 25,000 live male births respectively. Both Hemophilia A and B are X-linked genetic disorders. The severe phenotype of both forms is defined as the production of FVIII and FIX less than 1 IU/dL. Patients with severe hemophilia are susceptible to life-threatening severe bleeding during medical aggressive conditions such as trauma and surgery. The somewhat effective treatment for severe hemophilia is the regular application of FIX/FVIII to elevate the blood FIX/FVIII above 1 IU/dL [81]. With advances in GT approaches, the number of clinical trials on this disease during the last decade has risen. In 2014, A.C. Nathwani et al. published the results of GT on severe Hemophilia B utilizing AAV8. Out of 10 patients, 6 received high doses, 2 received intermediate doses, and 2 received low doses of AAV2/8-LP1-hFIXco (AAV encoding FIX). The FIX level after AAV-based GT increased to 1–6% of the normal level depending on the dose of AAV2/8-LP1-hFIXco. In the high-dose group, the FIX level increased to 5.1 ± 1.7 %, which resulted in a decrease in FIX application. Moreover, all 10 patients experienced a decrease in annual bleeding rate. The most frequently observed SAE was the elevated alanine aminotransferase (ALT) level [82] (Table 2, Row No.14). After an interval of 3 years, the same results were applicable to another trial published by Savita Rangarajan et al., in 2017. In this study, 9 male patients with severe hemophilia A were divided into three dose-based cohorts (Low, intermediate, and high doses) and were treated with valoctocogene roxaparvovec-rvox (FDA-approved AAV5-based GT product). One patient received a low dose, one patient received an intermediate dose, and all other patients received high-dose. The results indicated that the patient who received a low-dose vector had FVIII activity level under 1 IU/dL. The patient who received an intermediate-dose vector had, although low, stable FVIII activity. The results were more favorable for the high-dose cohort [83] (Table 2, Row No.15). In 2020, Steffen Rosen et al. published an update of this trial aiming to compare the activity of transgene-produced FVIII-SQ with recombinant FVIII-SQ. The study found that FVIII-SQ measured by the one-stage clot (OS) assay showed higher activity than when measured by the chromogenic-substrate (CS) assay, in contrast to recombinant FVIII-SQ which had lower activity in the OS assay. Despite these differences, both transgene-produced and recombinant FVIII-SQ showed similar specific activity when tested using the CS assay. Additionally, the study revealed that transgene-produced FVIII-SQ triggered a faster onset of factor Xa and thrombin formation, which could explain the higher activity readings in the OS assay. These results provide important insights into the behavior and performance of transgene-produced FVIII-SQ compared to recombinant FVIII-SQ, and their clinical significance in treating hemophilia A [84] (Table 2, Row No.15). In 2017, another trial of GT on Hemophilia B had its results published. In this trial, 10 men with hemophilia B were included restrictively having ≤2 % of FIX. Following AAV-based GT, the FIX level increased to an average of 33.7 ± 18.5 % of the normal level with no SAE related to GT. Additionally, a reduction in the annual bleeding rate was observed, and FIX application decreased to the extent that some participants became independent of FIX-application. Elevated ALT level was observed as AE similar to 2 previous studies. Additionally, progression of the underlying disease was observed in patients who had arthropathy status as an AE preceding GT [85] (Table 2, Row No.16). During 2018, another GT trial on hemophilia B with 2 dose-based cohorts, low and high doses of AAV5 encoding FIX, showed that in both cohorts the FIX level increased significantly, while the bleeding rate and factor application faced a dramatic decrease. Significantly, elevated ALT levels were observed in both cohorts (1 patient in the low-dose cohort and 2 patients in the high-dose cohort) which is considered to be an AE. However, akin to other trials preceding that year, the elevation was short-term and promptly managed [86] (Table 2, Row No.17). In the same year, the long-term results of a GT trial were published by Lindsey A. George et al. in which 7 Hemophilia B patients underwent AAV2-based GT. No safety concerns were observed and no signs of persistent hepatic toxicity or hepatocellular carcinoma were evident, although the initial observations on animal models have shown these complications. From the beginning to the last follow-up of patients in this trial, high levels of neutralizing antibodies (NAbs) against multiple serotypes of AAV were evident for 15 years. [87] (Table 2, Row No.18). At this point in 2020, K. John Pasi et al. updated the results of the GT trial that Savita Rangarajan et al. published back in 2013 which corroborated the findings of previous studies on hemophilia patients. In this trial, the GT procedure utilizing AAV5 on 15 patients with severe hemophilia A showed that within three years after infusion, patients who received different doses of the treatment showed varied FVIII expression levels and bleeding event outcomes. Significantly, patients who received higher doses experienced increased FVIII expression and reduced bleeding events, even though some of them achieved complete resolution of bleeding in target joints. The treatment also showed stable FVIII expression and effective mirroring of the hemostatic ability of native FVIII. No AEs such as inhibitor development, thromboses, deaths, or persistent changes in liver-function tests were observed [88] (Table 2, Row No.15). In another study on hemophilia B, published in 2020 by Barbara A. Konkle, BAX 335 (A vector based on AAV8 encoding the hyperactive FIX named Padua variant) was administered to 8 adult male patients. Each participant received one of three intravenous dosage levels of BAX 335 (low, intermediate, and high doses). One participant achieved sustained therapeutic FIX activity without bleeding or replacement therapy for 4 years, while in others, FIX activity was not sustained beyond certain durations. The study also demonstrated that SAEs occurred, but were considered unrelated to BAX 335. Moreover, there were no signs of clinical thrombosis, or other FIX Padua–directed immunity. The loss of transgene expression was hypothesized to be caused by the stimulation of innate immune responses [89] (Table 2, Row No.19). In 2021, in an approach to treating Hemophilia A with SPK-8011 which is a rAAV, Lindsey. A. George and her team involved 18 patients in 4 dose-based cohorts, from 5 × 10^11^ vector genomes per kilogram (vg/kg) of body weight to 2 × 10^12^ vg/kg. The results of this trial showed that the expression of FVIII was completely lost in 2 patients due to a cellular immune response against the AAV's capsid, which was unresponsive to immunosuppressive treatment. FVIII expression remained consistent in the other 16 patients. In 12 of them who were followed for more than two years, the level of FVIII activity did not show any meaningful decline. In the absence of glucocorticoid treatment, the activity levels of FVIII remained at approximately 12.9 ± 6.9 % of normal between 26 and 52 weeks after GT. This was similar to the level of 12.0 ± 7.1 % of normal measured after 52 weeks post-treatment. Nonetheless, in this trial SAEs related to GT and its product were found [90] (Table 2, Row No.20). In 2022, two GT trials were conducted on hemophilia B patients by Pratima Chowdary et al. and Feng Xue et al., both of which showed FIX increase and elevated aminotransferase as AEs [91,92]. In the trial held by Pratima Chowdary et al., 10 patients were divided into four dose-based cohorts (3.84 × 10^11^ vg, 6.40 × 10^11^ vg, 8.32 × 10^11^ vg, and 1.28 × 10^12^ vg). According to the results of this trial, the increase in FIX level depends on the dose of GT material that the patients receive and is more in those who received the highest dose. In this study, the arteriovenous fistula was GT-related SAE [91] (Table 2, Row No.21). Contrary to the previous trial, in which there were four dose-based cohorts, the results published by Feng Xue, stated that 12 male hemophilia B patients were screened, and an additional 10 were enrolled., all of which received a single injection of the GT product equal to 5 × 10^12^ vg/kg. The results of this study were favorable since the FIX activity level increased to an average of 36.9 IU/dL without SAEs [92] (Table 2, Row No.22). In the same year, another GT trial on 134 patients with severe hemophilia A who took one dose of Valoctocogene Roxaparvovec (An AAV5 encoding FVIII) revealed that the mean FVIII activity level for HIV-negative patients increased by 41.9 IU per deciliter. The FVIII application and managed bleedings also decreased after the infusion. All patients experienced at least one AE, with 22 reporting SAEs. Most common AEs included elevations in liver enzyme levels, which were managed with immune suppressants, as well as headaches and nausea. However, no participants developed FVIII inhibitors or thrombosis [93] (Table 2, Row No.23). In 2023, J. Mahlangu et al. published the new results of this trial, which showed that after treatment with AAV encoding FVIII, the bleeding rate decreased, and no SAEs or safety concerns were reported. However, starting from the 76th week, the decline in the activity level of the FVIII protein produced by the transgene in the body exhibited a consistent pattern characteristic of first-order kinetics. [94] (Table 2, Row No.23), In the same year, S.W. Pipe et al. published the results of a GT trial utilizing Etranacogene Dezaparvovec, which is a FDA-approved AAV5-based GT product, on 54 patients in which the Padua variant of human FIX was introduced. Included patients showed a decrease in the bleeding rate and FIX application and an increase in FIX activity. Additionally, this approach was considered safe since there were no SAEs related to the GT product [95] (Table 2, Row No.24). As far as this review is concerned, AAVs are the most frequently used vectors in Hemophilia GT, and with the wealth of information provided by these clinical trials, it can be concluded that GT for both Hemophilia A and B, although not AE-free, is feasible and can at least alleviate the complication's associated with this particular disease. However, future investigations should focus on achieving long-term expression of FIX or FVIII and increasing GT's impact on reducing the bleeding episodes over the long term.

## 4 FDA-approved GT products

There are many products with RV, LV, AAV, and other vectors that have been approved by the FDA, which some of them were related to the treatment of immune deficiencies and blood disorders. Table 2 displays a list of viral-based GT products approved by the FDA up to the time of writing this article. The table includes important information for each product, such as the product's commercial name, the kind of viral vector it uses, the specific illness it treats, the date it was approved, and any useful clinical advice or instructions for use. This table is handy for medical workers, scientists, and patients who want to stay informed about the latest developments and official approvals in GT (Table 3).

**Table 3:** FDA-approved GT products.

Proper Name	Tradename	STN	Manufacturer	Disease	Vector	Date of approval	Price	Link
Valoctocogene roxaparvovec-rvox	ROCTAVIAN	125,720	BioMarin pharmaceutical Inc.	Hemophilia A	AAV	June 29, 2023	$2.9 million	
Etranacogene dezaparvovec-drlb	HEMGENIX	BLA 125772	CSL behring LLC	Hemophilia B	AAV	November 22, 2022	$3,500,000	https://www.fda.gov/vaccines-blood-biologics/vaccines/hemgenix
BREYANZI	BREYANZI	BLA 125714	Juno therapeutics, Inc., a bristol-Myers Squibb Company	Large B-cell lymphoma (LBCL)	LV	June 24, 2022	$470,939.53	https://www.fda.gov/vaccines-blood-biologics/cellular-gene-therapy-products/breyanzi-lisocabtagene-maraleucel
Brexucabtagene autoleucel	TECARTUS	BL 125703	Kite pharma, Inc.	MCL/ALL	RVl	Oct 1, 2021	$373,000	https://www.fda.gov/vaccines-blood-biologics/cellular-gene-therapy-products/tecartus-brexucabtagene-autoleucel
Betibeglogene autotemcel	ZYNTEGLO	125,717	Bluebird bio Inc.	β-thalassemia	LV	Aug 17, 2022	$2,800,000	https://www.fda.gov/vaccines-blood-biologics/zynteglo
Axicabtagene ciloleucel	YESCARTA	BL 125643	Kite pharma Inc.	B-cell lymphoma	RV	April 1, 2022	$373,000	https://www.fda.gov/vaccines-blood-biologics/cellular-gene-therapy-products/yescarta-axicabtagene-ciloleucel
Ciltacabtagene autoleucel	CARVYKTI	125,746	Janssen biotech, Inc.	Myeloma	LV	Febuary 22, 2022	$465,000	https://www.fda.gov/vaccines-blood-biologics/carvykti
Idecabtagene vicleucel	ABECMA	BLA 125736	Celgene Corporation, a bristol-Myers Squibb Company	Multiple myeloma	LV	March 26, 2021	$419,500	https://www.fda.gov/vaccines-blood-biologics/abecma-idecabtagene-vicleucel
Tisagenlecleucel	KYMRIAH	125,646	Novartis pharmaceuticals Corporation	Relapsed or refractory follicular lymphoma.	LV	August 30, 2017	$475,000	https://www.fda.gov/vaccines-blood-biologics/cellular-gene-therapy-products/kymriah-tisagenlecleucel
Onasemnogene abeparvovec-xioi	ZOLGENSMA	125,694	Novartis gene therapies, Inc.	Spinal muscular atrophy (SMA)	AAV	May 24, 2019	$2,125,000	https://www.fda.gov/vaccines-blood-biologics/zolgensma

∗Although it is not in the timeline target of this paper, it is worth mentioning that as of the time of the publication of this article (2024), another GT product has gotten FDA approval, fidanacogene elaparvovec. This is a AAVRh74var-GT product to cure Hemophilia B.

## 5 Discussion

GT is the most promising cure for many diseases that used to be known as incurable for years [96]. Concerning desired genetic material delivery, there are two main delivery systems, viral and non-viral-based vectors. Non-viral vectors are associated with advantages and disadvantages. The main advantages of these vectors are as follows: minimal immune reactions, non-toxic nature, and being less expensive [18]. These vectors, however, are associated with some major concerns such as safety, specificity, the duration of gene expression, and the efficiency of gene transfer. On the other hand, viral-based GT is an efficient approach because its nature is optimized to infect living cells, and the transgene is highly protected within the vector genome [18]. These vectors are also more efficient than non-viral vectors in gene delivery [18]. Each viral vector has a unique tropism, for instance, most AAV viruses are hepatocyte tropic, RV viruses are tropic to dividing cells, and LV viruses are tropic to both non-dividing and dividing cells. The choice of a proper vector for GT depends on various factors including the tissue to be targeted, the vector's packaging capacity, and the way of introducing therapeutic genes into target cells [15,17,[41], [42], [43],^50^,^79^,^92^]. For example, the most frequently used vectors among hemophilia GT trials in the last decade are AAV5, AAV8, and AAV2 respectively, resulting from their tropism [68]. Despite the benefits offered by viral vectors, concerns remain due to their potential immunogenicity and cytotoxicity, as well as the high costs associated with their production [2,89]. Moreover, there are some concerns about the insertional mutagenesis associated with RVs, and carcinogenesis of AAVs in mice in long-term observation. As for the carcinogenesis risk of AAVs, nevertheless, the results are controversial, so further studies with this regard is highly suggested [97]. However, all these concerns lead to the development of recombinant viral vectors in which the risk of any major AE is reduced [90].

This review primarily focuses on the outcomes of GT in SCID, WAS, CGD, LAD, hemoglobinopathies, FA, and hemophilia. These disorders, except hemophilia, are widely recognized for having allo-HSCT as a curative treatment [21,41,44,52,53,60]. HSCT from full-matched related donors is the best form of HSCT. However, this approach is still associated with risks. There is a risk of developing GvHD in patients undergoing HSCT. Immunosuppressive conditioning related to HSCT may also introduce the risk of toxicity in patients. GT provides a safer treatment for patients who have allo-HSCT as their only chance to be cured since the risk of GvHD is not a concern for patients and there is no need for immunosuppressive therapy afterward [23,41]. Generally, LV and RV vector-based GTs have consistently involved the use of autologous HSCT following ex vivo genetic modification in trials conducted over the past ten years. In contrast, in in-vivo GT, such as those using AAV vectors, HSCT is not required [30,36,43,66]. The most recent GT studies on immune deficiencies and blood disorders which are discussed in this review showed significantly favorable results and every trial had unique information that could push the investigation one step closer to pure success regarding viral-based GT. For instance, GT for SCID showed that RV-based GT might elevate the risk of leukemia development, but it can also benefit patients with immune reconstitution and ERT discontinuation. Leukemia was also seen in WAS GT as SAE, but it was one of the first GT trials on this disease. Recent advances in GT for CGD have yielded positive results and could change the bias of GT in this disease formed as a consequence of previous failures, although immune reconstitution inflammatory syndrome was evident as an AE related to CGD GT. Research into GT for treating LAD has been quite limited over the past decade, resulting in an unclear understanding of both its efficacy and potential side effects in LAD patients. Considering that LAD-1 might be more prevalent than estimated in certain countries, it is crucial to conduct further investigations into the efficacy of GT, with a particular focus on viral-based methodologies, for this disease. GT in hemoglobinopathies has also shown many favorable outcomes but the most significant outcome is a TDT patient that was infected with wild-type HIV during the LV-based GT which might be a result of the transformation of rLV into its wild-type. Nonetheless, this result alone may not be extended to all GTs on TDT patients, because no other patient was reported to be infected with HIV in any other trials afterward. FA GT didn't prove any AEs related to the GT approach and was successfully accomplished up until the date of writing this article. In hemophilia patients in whom HSCT is not an option, GT can relieve them from regular factor application, which is the only treatment to hinder the disease symptoms [68]. Hemophilia GT was full of exceptions since the vector differed from other diseases discussed, for example, in hemophilia gene therapy trials, patients were treated in different cohorts with varying doses of the gene therapy material, unlike other approaches for different diseases and the only common AE evident was the elevated ALT level. However, limited number of AEs are specific to the use of AAV in Hemophilia GT and can have many more AEs associated with the use of AAVs in the treatment of other diseases, such as acute liver failure, and hepatitis [98].

It is worth mentioning that each GT approach during the last decade had a conditioning regimen specifically related to the disease. For some diseases, an MA conditioning regimen was mostly considered, while for others, a reduced-intensity (non-MA) conditioning regimen was mostly designed. Nonetheless, ex-vivo GT appears to require the design of a conditioning regimen which is a result of the general concept of this type of GT, so AAV-GT may be able to mitigate the conditioning-related complications even compared to the approaches that use less aggressive conditioning like RIC. During the last decade, many GT products have gained FDA approval. However, because these products are quite expensive, they might not be accessible to the public. To recapitulate, with the evidence of 10 years of GT reports, it can be implied that the safety and efficacy of GT are highly dependent on the utilized vector and type of disease. In the future, as the use of non-viral vectors increases, it may reduce the risks and cost of GT. Additionally, the potential use of hybrid vectors, which combine non-viral vectors and genes associated with viruses for cell and tissue entry, could enhance the safety and effectiveness of GT. Noteworthy, most viral GT trials reviewed in this article had favorable results in all diseases and GT can be a safe and common approach for most inherited diseases in the future.

## 6 Conclusion

Up to the date of writing this review, viral-based GT has been having promising results in the treatment of immune deficiencies and blood disorders. Viral-based GT can be even safer in the future by utilizing the results and outcomes of previous trials. Moreover, with the development of technology and knowledge of each viral vector, GT can become the first common treatment in the near future.

## Authors’ contributions

SE, MMB, and EI contributed to paper writing and generated figures. MB contributed to the edition of medical content. RM and LJ contributed to the edition of viral content. PN contributed to the study design. AAH and ZS contributed to the data discussion and revised the manuscript.

## Availability of data and materials

Not applicable.

## Consent for publication

Not applicable.

## Ethics approval and consent to participate

Not applicable.

## Funding

Not applicable.

## Declaration of competing interest

The authors declare that they have no known competing financial interests or personal relationships that could have appeared to influence the work reported in this paper.
